# Do working conditions contribute differently to gender gaps in self-rated health within different occupational classes? Evidence from the Swedish Level of Living Survey

**DOI:** 10.1371/journal.pone.0253119

**Published:** 2021-06-15

**Authors:** Sara Kjellsson

**Affiliations:** 1 Swedish Institute for Social Research, Stockholm University, Stockholm, Sweden; 2 Department of Public Health Sciences, Stockholm University, Stockholm, Sweden; Universitat de Valencia, SPAIN

## Abstract

Socioeconomic inequality in health among women is often referred to as smaller than health inequality among men. However, we know less about differences in health between men and women within the same socioeconomic groups. In this article the lack of attention to potential socioeconomic variation in gender health inequality is argued as unfortunate, as it can obscure how mechanisms, such as e.g. working conditions, affect gendered health within specific groups. Drawing on the nationally representative Swedish Level of Living survey (LNU), class/gender interactions as well as class-separate linear probability models are estimated to explore relationships between working conditions and health among men and women with the same occupational class positions. Results show that, although class is not a large explanatory factor for general gender differences in health, there are varying within-class differences between men and women in working conditions, that can contribute to the understanding of within-class gender differences in health. This highlights that, when targeting causes of gender health inequality, it is important to consider not only what class means for women as well as for men, but also what gender means within specific classes.

## Introduction

According to previous research, inequality in health based on socioeconomic position (SEP) is larger among men than among women [1,2]. Other scholars have, however, disputed this as a universal find [3] and differing results could be connected to the health outcome [4], inequality measure [5], or SEP measure [6] used. As previous research mainly has paid attention to gender differences in the relationship between SEP and health, less is known about whether the relationship between gender and health varies by SEP [7,8, see however 9,10]. In this article, the lack of attention to a potential SEP variation in gender health inequality is argued as unfortunate. When we expect gender differences to be general (over SEP), there is a risk that we overlook specific mechanisms that are of importance for differences between men and women within a specific SEP.

Working conditions are potential mechanisms that can have impact on health and class is the SEP-measure most closely connected to working conditions [6]. Demanding working conditions are more common in working class than in white-collar occupations and has been found as a mechanism for class differences in health [e.g. 11–13]. The distribution of working conditions may also influence gender differences in health. From the literature on gender-segregated labour markets, we know that men and women are distributed differentially on the hierarchical axes of occupations, so called vertical gender segregation. Furthermore, men and women are typically both employed in different occupations, as well as perform different types of tasks when they are in similar occupations, so called horizontal gender segregation [14,15]. Working conditions for men and women can thus differ due to both axes of segregation and these differences can vary by class.

The aim of the present study is explorative, seeking to investigate the potential for class differences in the relationship between gender and health. Do gender differences in health vary by class? And can class-specific gender differences in physical or psychosocial working conditions further our understanding of the relationships between gender and self-rated health problems?

### Working conditions and health

Physically demanding working conditions entail physical demand or strain and may thus primarily be expected to affect physical health. Conditions such as e.g. heavy lifting and repetitive, forceful, static or otherwise unsuitable movements or positions can result in wear and tear of the body and thereby cause aches and pains [16]. Psychosocial working conditions (referring to both the psychological and the social aspects) have also been linked to ill health. The concept *job strain* refers to a combination of mentally demanding work with low possibility of controlling your work situation [17]. Several studies have linked job strain to stress as a risk factor for psychological ill health, but there is also a discussion about the relative importance of the joint vs. the independent role of demand and control [for review, see 18]. Some studies have found larger impact of psychosocial conditions on health among men than among women [19]. Emotional requirements have also been suggested to be included in *job strain* as demanding working conditions [20]. Even if emotional investments at work may not be harmful per se, such requirements can cause strain in a situation where you experience low control [21]. Additionally, long-term exposure to stress can lead to muscle tension and subsequent bodily pain [16,22].

Another potential contributor to stress is a lack of possibility to combine family- and work-life demands, and flexible jobs that easier accommodates family obligations are sometimes described as “mother-friendly”. However, in Sweden, female dominated occupations do not seem to entail more flexibility than male dominated occupations [23]. Evidence from Germany also suggest that flexible work arrangements may involve increased productivity demands for women but not for men [24], which could imply larger risks of job-related stress for women compared to for men. However, recently, a group of researchers in the UK failed to find a relationship between either work time flexibility or work place flexibility and stress, but they did find a negative association between reduced working hours and stress [25].

### Class, gender and working conditions

Class-based health inequality has been noted to differ between men and women [1,2] but gender-based health inequality could also differ between classes [cf. 9,10]. Some scholars have raised the concern that class schemas are constructed in relation to a male labour market and therefore do not explain or predict conditions among women equally well, which would be one reason why class inequality is measured as smaller among women than among men [3]. On the other hand, it has been suggested that in societies where the share of women on the labour market is large, occupationally-related stratification will be a more salient factor for differences also among women [26,27]. Empirical studies have shown that the class concept is equivalent for men and women in regards employment relations [28] as well as long-term economic outcomes [29]. However, that the class concept is equally applicable does not imply that men and women are distributed equally within the class structure.

Most labour markets are segregated by gender, something that can be described as running both “vertically” and “horizontally” [14,15]. *Vertical gender segregation* refers to an underrepresentation of women in the top of status hierarchies, such as at higher SEP. That conditions (resources, opportunities, rewards) differ along the hierarchical dimension is an underlying tenet of social stratification. When it comes to working conditions and class, working class occupations typically involve both more manual labour and less autonomy than service class occupations [30,31]. Working class occupations are thus characterized by harder physical work as well as less opportunity to control the work situation. *Horizontal gender segregation* refers to work content, where occupations and/or tasks are socially coded on a scale from masculine to feminine. This can lead to gender differences in working conditions if male and female coded occupations involve different conditions. Physical labour can be expected to involve the need for physical strength (such as heavy lifting, operation of heavy machinery), which is conceptually connected to masculinity [32,33] and physically strenuous working conditions is a common feature of traditionally male working-class occupations, e.g. within the production industry. However, work that involves human interaction, such as e.g. interpersonal care also contains physical tasks [15] and physical work is common in traditionally female occupations such as health-, elder- or childcare [34]. Emotional work is found in occupations within e.g., social work, nursing, teaching, and eldercare. Such occupations are often female dominated, but many of these are not working-class occupations and it would thus not affect class differences–only the gender difference. Since the two types of gender segregation co-exist, working conditions can differ between men and women due to both vertical and horizontal segregation. Furthermore, if the level or nature of horizontal gender segregation is different along the vertical axis it is possible that the gender difference in working conditions varies by SEP.

There is quite considerable gender segregation on the Swedish labour market, men and women largely work in different occupations and with different tasks [15,35]. The share of women within higher-level occupations is lower than the share of men but the vertical dimension of gender segregation has become less evident over time, as women occupy an increasing share of high-ranking positions [36]. Horizontal segregation, on the other hand, has changed at a slower pace. Horizontal segregation also seems to be more pervasive at the bottom of the vertical hierarchy, with less prestigious occupations being more socially gendered than higher status occupations [15,36]. The expectation here is thereby of larger gender differences in working conditions within the working class, and as such larger scope for differences in ill health.

## Data and method

This study uses the 2010 wave of the Swedish Level of living survey (*Levnadsnivåundersökningen* [LNU]). LNU is a longitudinal survey based on a random sample of 1/1 000 of the Swedish population age 15–75 in 1968 (the age-span was changed to 18–75 in 1991). Refresher samples have been added in each additional wave of the study, making each wave also nationally representative as cross-sectional data (www.sofi.su.se; SCB n.d.). The sample in 2010 consisted of 7 253 individuals, whereof 4 415 answered the survey, giving a 60.9% respondent rate (see Fig A in S1 Appendix). The response rate was slightly larger among women (61.4%) than among men (60.0%) (SCB n.d., Tabellbilaga B, pp. 2) and it was also lower in the age groups below 30 (53.0–58.0%) (SCB n.d., Tabellbilaga B pp. 5). LNU was approved by the Regional Ethical Review Board in Stockholm (see www.etikprovningsmyndigheten.se) and all research is carried out on de-personalized data. Consent is obtained from each survey participant at the time of the interview.

The study sample was restricted on three criteria, all in reference to the focus on work conditions: i) Age 18–65. Individuals working after the regular retirement age can be expected to be selected both on health and on class, therefore 65 years of age was set as an upper age limit for inclusion, resulting in the exclusion of 636 respondents. ii) To be in employment. Respondents were asked if they were employed full time and if they were employed part time during the week prior to the interview and respondents that answered “No” on both were excluded from the study (n = 1 128). iii) To have available information on work conditions. Employers and those in self-employment (incl. farmers) are not asked about working conditions in the LNU surveys and were therefore not included in the study (n = 14). (However, as the questions on full- and part time employment specifically refer to being in someone else’s employ, most self-employed and employers were already excluded). further 40 respondents were excluded from the sample due to missing information on independent variables.

The final study sample includes 2 597 individuals which represent 68.7% of the respondents in the age group 18–65 (69.8% before exclusion on missing variables). According to official statistics, the share of employed individuals in the Swedish population (age 16–64) in year 2010 was 65% [37]. Given that Swedish secondary education is finished around age 18–19, a slightly lower share of employees in the age group 16–64, compared to 18–65, is expected. The share of men (51.4%, n = 1 335) and women (48.6%, n = 1 262) in the study sample of employed 18 to 65-year-olds is further similar to the shares in the employed population of 16 to 64-year-olds (50.2 and 49.8% respectively).

The included variables are described below, and the variable distribution can be found in the Appendix (Table A in S2 Appendix).

### Health variables

Self-rated health (SRH) is a common health measure in surveys. It is a stable measure of individual health, a good predictor of subsequent mortality [38,39] and gives a broad picture of the individual’s experience of health [40]. In LNU, respondents were asked to rate their overall health as either “good”, “bad”, or “something in-between”. Since the article concerns differences in the experience of ill health, the answers were dichotomised into a dummy variable for less than good SRH (*bad/something in between* = 1).

Regarding the more symptom specific health measures included, musculoskeletal pain is a health problem shown to be more common among women than among men [41,42] and has been identified as one of the major causes of sickness absence from work in Sweden, particularly for women [43]. Psychological distress is generally found to be more common among women than among men, while the results for socioeconomic differences are somewhat mixed [44,45]. Rising levels of distress among women has also been a cause for concern within the Swedish political debate during the early 21^st^ Century [46]. The LNU-survey contains a list of various health symptoms and the respondents are asked to state which symptoms or problems they experienced during the last 12 months and whether they were experienced as mild or severe. Three symptoms on this list refer to musculoskeletal pain: pain in the neck and shoulders; pain in the back, hips and/or sciatica; pain in the joints. Five symptoms refer to distress: tiredness, sleeping problems, anxiety, depression, and overexertion. One dummy variable each were constructed for having experienced i) at least one symptom of musculoskeletal pain and ii) at least one symptom of distress. Respondents who had experienced at last one of the relevant symptoms (mild or severe) were given the value = 1 (*otherwise = 0*). Different specifications of these variables have been tested and no substantial differences in results or interpretations were drawn.

### Working conditions

If the respondent reported both a full time and a part time employment, the working conditions refers to their full-time position and respondents with multiple part time jobs (and no full time) reported working conditions for the position that they defined as their primary employment.

For physically strenuous conditions, respondents were asked if their job requires them to lift > = 60kg and, if so, how often this is required. In order to capture heavy lifting on a regular basis the answers “daily”/ “a few times a week” were here coded = 1 (*more seldom/no = 0)*. The respondents, furthermore, stated whether their jobs are “otherwise” physically demanding; makes them sweat from physical exertion; forces them into unsuitable work positions; involves many repetitive and monotonous movements, and whether they sit down most of their working time (*yes = 1*, *no = 0*). A factor analysis of these physically strenuous conditions turned out one factor with eigenvalue >1 that included heavy lifting, physically demand, daily sweating, and unsuitable positions (all factor loadings >0.60, except heavy lifting = 0.42). These four items were thus summed to an index of physically strenuous work (0–4) with a Cronbach’s Alpha of 0.73 (excluding heavy lifting had a negligible effect, Cronbach’s Alpha = 0.74). To sit down most of the workday loaded in the opposite direction to the other items (-0.61) and was therefore used as a separate dummy variable (*yes* = 1) in analyses. The question of repetitive movements was also included as a dummy variable (*yes* = 1) since it did not load highly in the chosen factor.

Job strain was defined in accordance with Karasek and Theorell’s [17] original model. Respondents that are not able to control the pace of their work and that have monotonous tasks are considered to be lacking in control. Experiencing the job as stressful and mentally taxing is considered demanding work. The combination of lacking control and demanding work was then used as a dummy variable for job strain.

For emotionally demanding work, the respondents were asked how much working time that consists of such tasks. Answers were on a scale 1 (= *no time*, *or very seldom*) to 5 (= *all or almost all of the time*) where “half of the time or more” (> = 3) was coded = 1 for the dummy specification.

Work flexibility variables were based on yes/no questions about whether the respondent can decide when the workday should start/end, or whether the respondent can leave the workplace for half an hour to go on a private errand “without informing supervisor”. These were coded as dummies (*no* = 1) for non-flexible hours and for being without possibility for private errands.

Lastly, respondents were asked, on a scale from 1 (= *to a very large extent*) to 5 (= *not at all*), to what extent they can “get support and help from work-mates when needed”. The variable lack of social support at work was coded = 1 for only being able to receive support either “to a small extent” (4) or “not at all” (5).

### Gender

Information about whether the respondents are male or female comes from the official Swedish personal identification numbers (*personnummer*). Women were coded = 1 and make up 48.6 percent of the respondents.

### Class

In this study, SEP was measured as occupational class. There are various measures of SEP, such as education, occupational class or income. These are both interrelated and can have different and independent associations with health [6]. However, occupational class is the measure most closely connected to working conditions. Information about the respondent’s current occupation was coded according to the Swedish classification *Socio Ekonomisk Indelning* (SEI), which bears close resemblance to the internationally well-known Erikson-Goldthorpe class schema [47].

The categories used were unskilled workers (e.g. truck drivers, cleaners, shop assistants, personal care workers), skilled workers (e.g. carpenters, builders, child-care workers, restaurant wait staff), assistant non-manual employees (e.g. various clerks, administrative secretaries, police officers), intermediate non-manual employees (e.g. mechanical engineers, teachers, nurses) and higher non-manual employees (e.g. academic professionals, directors, executives). Dummy variables for each class category were constructed, and higher non-manual employees was used as reference category in all regression models.

Within the classes, there are notable differences in the types of occupations that are held by men and by women (a list of the top-five occupational groups for men and women, by class, is presented in Table B in S2 Appendix). Men’s occupations typically involve machinery, technology, computers and management, while women work with health care, personal care, teaching, and administration. There is also lesser variation in the occupations held by women, especially in the working classes. For example, the majority of female skilled workers are employed in personal care occupations (>70% including child-care).

### Interaction terms

Interaction terms between gender and each class category were constructed to test for class-specificity of gender differences. Male*Higher non-manual employees was used as reference category in regressions.

### Controls

All models were controlled for **age** (continuous) and **age squared** was included to capture possible acceleration of health deterioration at older ages.

### Analytic strategy

Analyses were estimated with Linear Probability Models (LPM), using Stata 14 software. LPM are similar to OLS-regressions run on a binary outcome, where coefficients are interpreted as percentage point’s change in the probability of the outcome. The results thus give the percentage point’s change in the probability of ill health (i.e. less than good SRH, at least one symptom of pain, at least one symptom of distress), given the included variables. Using LPM means assuming linearity on a binary outcome and a common choice for analyses of binary variables has been to use logistic regression. However, this issue has been discussed and it has been shown that when exploring relationships between variables and comparing coefficients between models, a logistic specification is less suitable [48,49]. LPM is therefore the preferred choice here. The focus in this study is, furthermore, to investigate patterns of health differences between the sexes rather than predicting specific probabilities.

The analytical strategy was divided into two steps, which were carried out for each of the three health outcomes. In step one, a baseline relationship between gender and health was estimated (unadjusted model). Then the mutually adjusted relationship between gender, class, and health was then estimated (Model 1) and interaction terms between gender and each class were added, to explore whether gender differences in health differ by class (Model 2). Predicted margins were then estimated based on model 2.

In step two, class-separate models were estimated to investigate whether working conditions can account for gender differences in health within each class. In this step, the unadjusted baseline model again included gender. Physically demanding working conditions (Model 1) and psychosocially demanding working conditions (Model 2) were then added stepwise.

All models were controlled for age and age^2. Throughout, 95% confidence intervals, based on robust standard errors, are reported.

## Results

**Fig 1:**
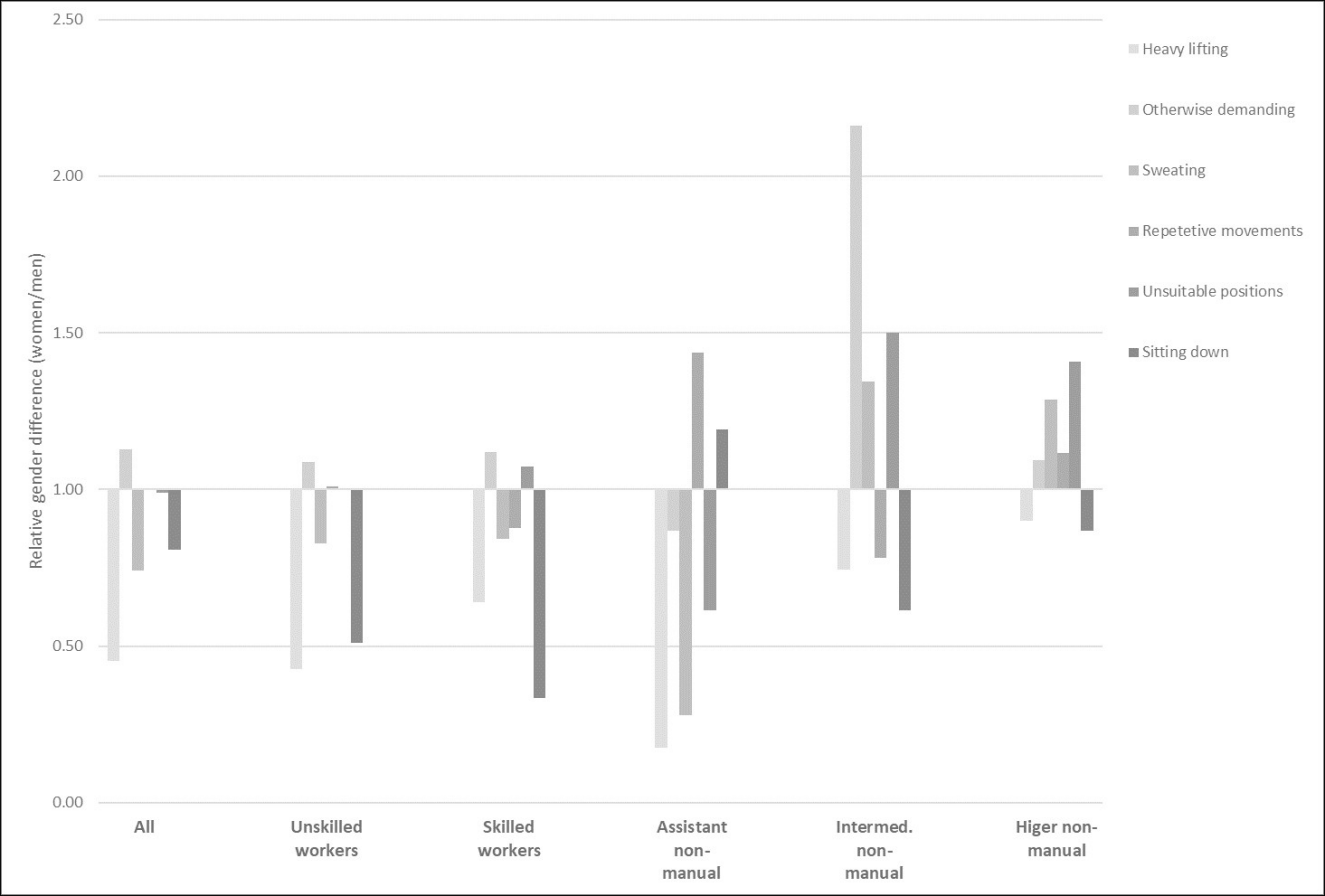
Gender differences in physically strenuous work, by class. Relative differences (percentage share women/percentage share men). Differences >1 indicate that the condition is more common among women than among men, differences <1 indicate that the condition is more common among men than among women.

The descriptive results show that, in general, it is more common among men than among women to have jobs that involve heavy lifting, that cause sweat, and that entails sitting down most of the time. Women, on the other hand, have “otherwise” physically demanding work to a larger degree than men, while repetitive movements and unsuitable positions are quite equally distributed between men and women (Fig 1). However, the gender difference in physically demanding working conditions varies between the classes. Among both intermediate and higher level non-manual employees, the difference in physical demand is more to the disadvantage of women, compared to in other classes.

**Fig 2:**
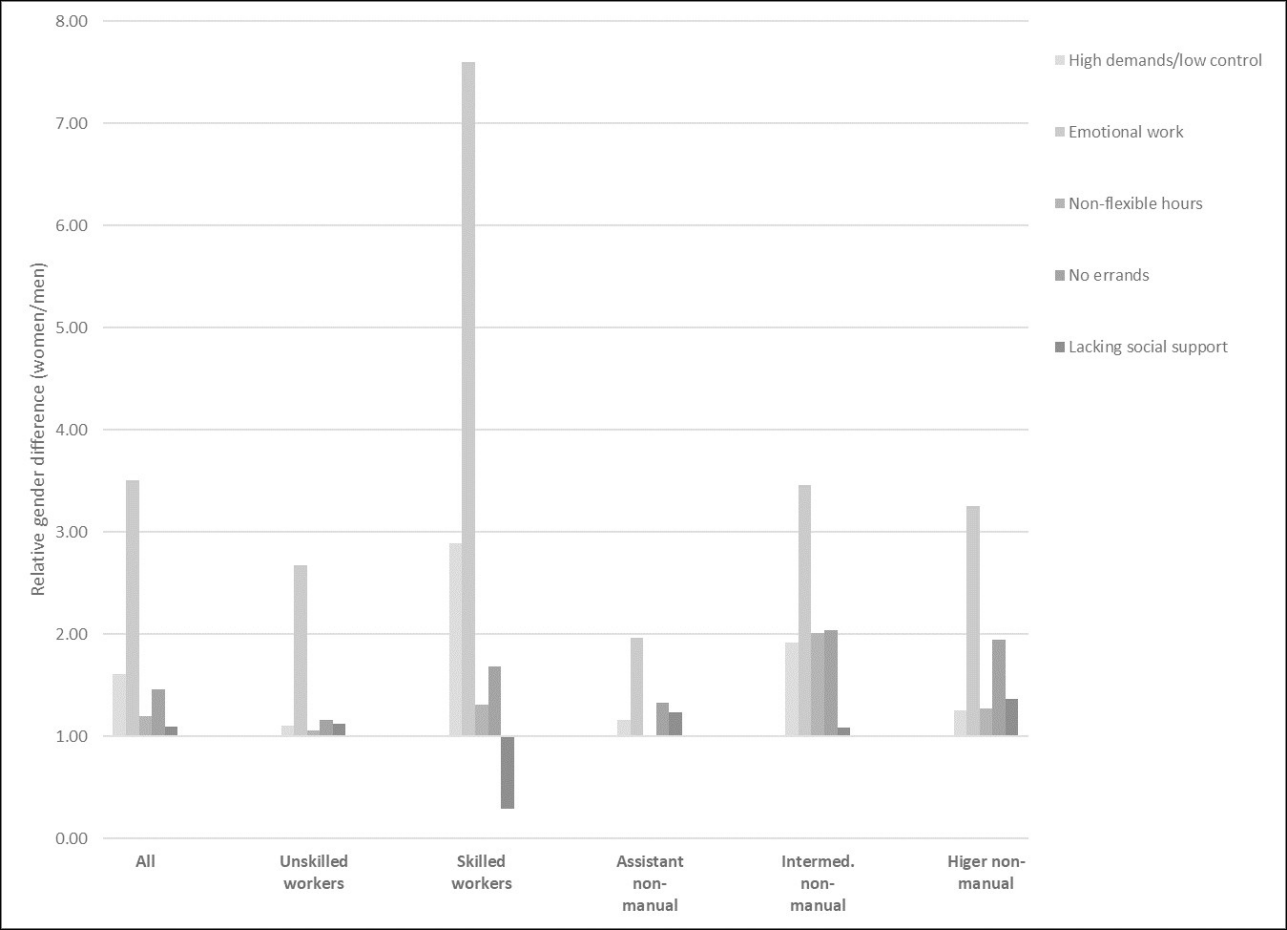
Gender differences in psychosocially demanding work, by class. Relative differences (percentage share women/percentage share men). Differences >1 indicate that the condition is more common among women than among men, differences <1 indicate that the condition is more common among men than among women.

Psychosocially demanding work is more common among women than among men and this pattern is found for practically all classes and all conditions (Fig 2). Overall, female skilled workers experience a high level of psychosocially demanding work, particularly compared to men within their class. The relative gender difference in job strain among skilled workers, shown in Fig 1, is almost 8-fold, which refers to that just under 50 percent of the women in this class experienced job strain, compared to 16 percent of the men (Table A in S2 Appendix). The gender difference in psychosocial demand is large also among intermediate non-manuals. For instance, female intermediate non-manuals work with emotional tasks 3.5 times more often than their male class-colleagues, and they experience job strain and non-flexible work conditions twice as often.

The share of women in the material, that experienced ill health is larger than the share of men, for all three health outcomes (Table A in S2 Appendix) and the general probability is 6 percentage points larger among women than among men for SRH, 8 percentage points for musculoskeletal pain and 16 percentage points for psychiatric distress (Table 1, Unadjusted). However, the gender differences are not greatly affected by the adjustment for class (Table 1, Adjusted). Thus, the general health disadvantage for women in SRH, pain and distress cannot be ascribed to class.

**Table 1:** Difference in probability for women, compared to men, to have experienced SRH, musculoskeletal pain and psychiatric distress.

	Unadjusted^1^		Adjusted^2^	
	coef.	95% CI	coef.	95% CI
**Self-rated health**	0.062	0.032 / 0.092	0.063	0.033 / 0.093
**Musculoskeletal pain**	0.084	0.046 / 0.122	0.087	0.049 / 0.126
**Psychiatric distress**	0.166	0.130 / 0.201	0.161	0.125 / 0.197

^1^Coefficients from unadjusted models regressing each health outcome on gender.

^2^Coefficients from models adjusted for class. Class was entered as dummy-coded variables for the categories of unskilled working class, skilled working class, assistant non-manuals and intermediate non-manuals, using higher non-manuals as reference category.

Coef. = coefficients from LPM-models, representing percentage points change in the probability of the outcome.

CI = Confidence interval.

Both unadjusted and adjusted models are controlled for age and age^2. Full linear models shown in Tables C–E in S3 Appendix.

Coefficients from LPM-models.

However, differences can be observed between the classes regarding the relative sizes of female/male reports of ill health and the patterns are somewhat different depending on the health measure used (Table A in S2 Appendix). For SRH the difference in the share of women, compared to the share of men, that reported ill health is less prominent among intermediate and higher-level non-manuals, while the largest relative difference is found among unskilled workers. The largest relative difference for musculoskeletal pain is instead found among assistant non-manual employees, and the smallest among unskilled workers. For psychiatric distress, the sizes of the gender differences in reported symptoms are similar across classes, with the exception of a smaller gap among higher non-manuals.

When allowing for the interaction between gender and class in the linear probability models, some class-specific variation in the gender health gaps can be observed. Although few of the interaction terms are statistically significant, there are some notable differences with larger gender gaps in SRH among the working class, and among assistant non-manual employees in musculoskeletal pain (Tables C–E in S3 Appendix). Below, the class-specific gender gaps in each of the three health outcomes, and their potential relationships with working conditions, are further investigated.

### Class-specific gender gaps in self-rated health

**Fig 3:**
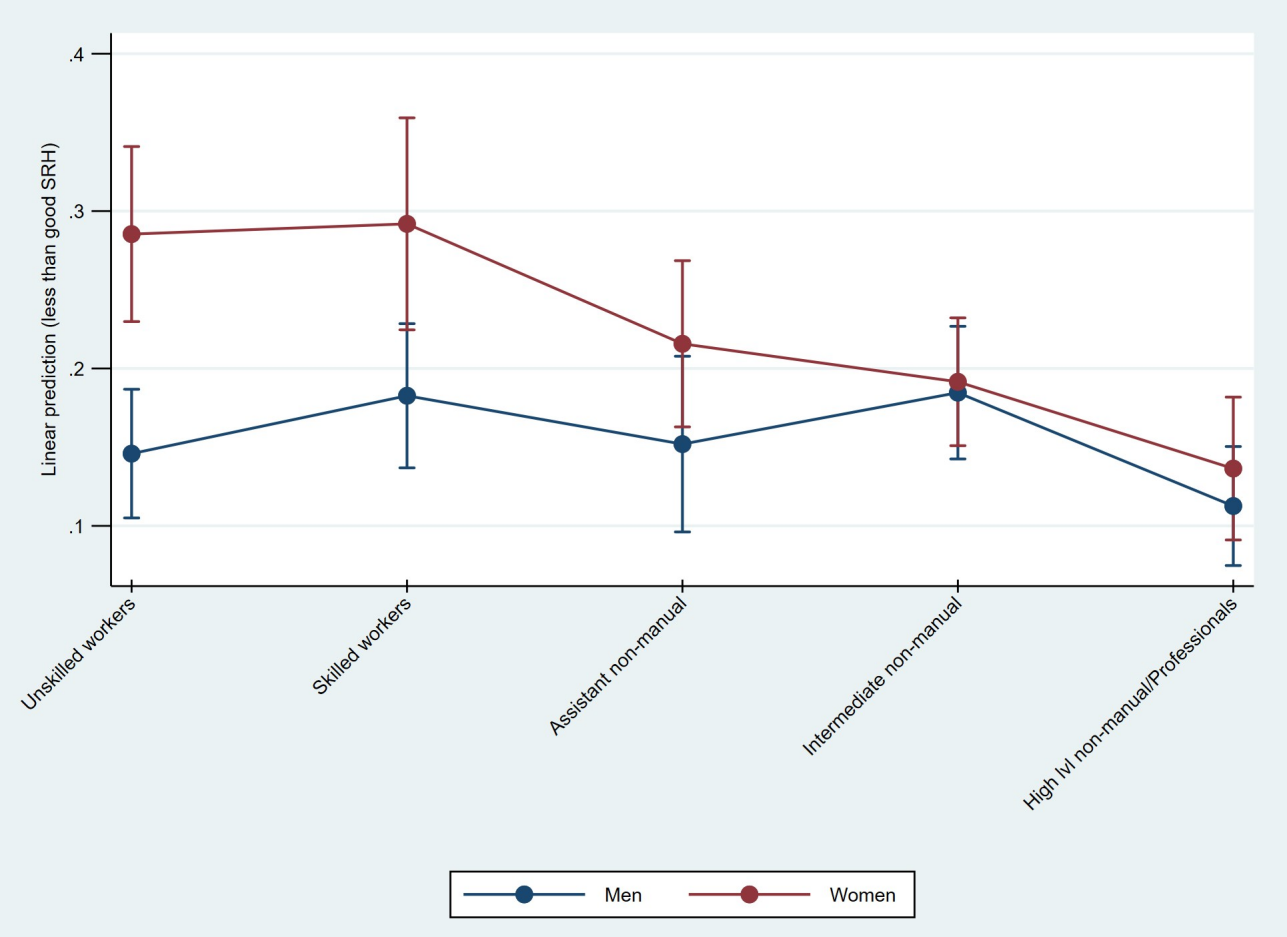
Predicted probabilities of less than good SRH, by gender and class. Marginsplot based on linear probability model with mutual adjustment for gender and class (Table C in S3 Appendix).

Predicted probabilities from the LPM show that the larger gender gaps within the working classes, compared to among non-manual employees, reflect higher predicted probabilities of less than good SRH for working class women, compared to both working class men and to women in the other class categories (Fig 3). For non-manual employees, the confidence intervals show substantial overlap and for employees at intermediate and high levels the predicted probabilities for men and women are essentially the same.

**Fig 4:**
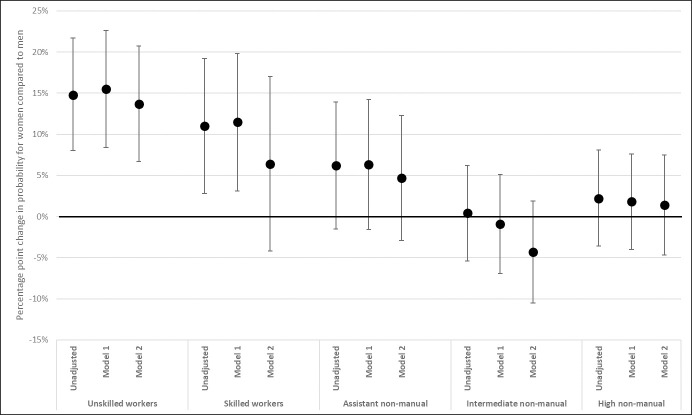
Gender differences in probability of less than good SRH, by class. Coefficients (for woman = 1) interpreted as percentage point change in probability for less than good SRH. From class-separate linear probability models, with 95% confidence intervals (Table F in S4 Appendix). Unadjusted model regresses SRH on gender, separately by class. Stepwise adjustment for physical working conditions (Model 1) and psychosocial working conditions (Model 2). Both unadjusted and adjusted models are controlled for age and age^2.

To explore the role of working conditions for the class-specific gender gaps in SRH, linear probability models were also estimated separately by class, with stepwise inclusion of physical and psychosocial working conditions. Within the unskilled working class, neither the inclusion of physical nor psychosocial working conditions affected the gender gap in SRH to any large extent (Fig 4). The point estimate for unskilled workers is slightly attenuated by the inclusion of psychosocial conditions while it increases somewhat when controlled for physical conditions. For the skilled working class there is a similarly small increase in the gender gap when including physical working conditions. However, psychosocial demand substantially attenuates the gender gap among skilled workers; the increase in predicted probability for women is almost halved, from 11 to 6,4 percentage points, indicating that psychosocial working conditions can explain part of the gender gap in SRH among skilled workers.

The case of intermediate non-manual employees, furthermore, present an exception to the pattern among the other classes. Here the inclusion of psychosocial working conditions reverses the gender gap, giving a predicted probability for less than good SRH that is almost 5 percentage points lower for women than for men. However, the point estimates are not statistically significant and should be interpreted with caution.

### Class specific gender gaps in musculoskeletal pain

**Fig 5:**
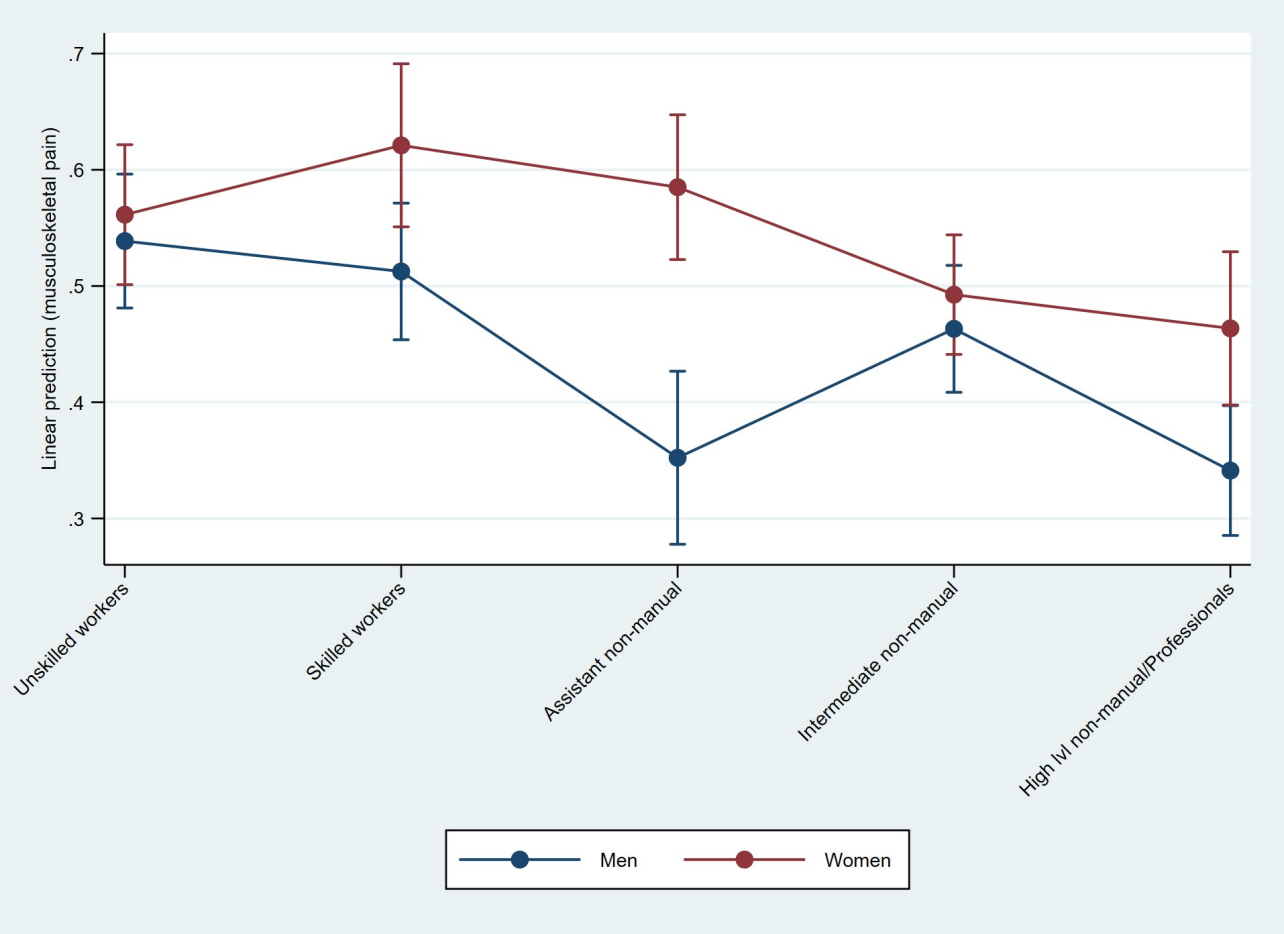
Predicted probabilities of musculoskeletal pain, by gender and class. Marginsplot based on linear probability model with mutual adjustment for gender and class (Table D in S3 Appendix).

The interaction between gender and class reveal no gender gaps among unskilled workers or intermediate non-manual employees and the predicted probabilities for musculoskeletal pain are quite equal for men and women in these classes (Fig 5). For skilled workers, assistant non-manuals and high-level non-manuals there are observable gender gaps but while the confidence intervals overlap for male and female skilled workers, the gender gap is particularly large among assistant non-manuals. The size of the gap among assistant non-manuals is driven by lower predicted probability of musculoskeletal pain among men in this class compared to both their female class-counterparts and compared to men in the other classes.

**Fig 6:**
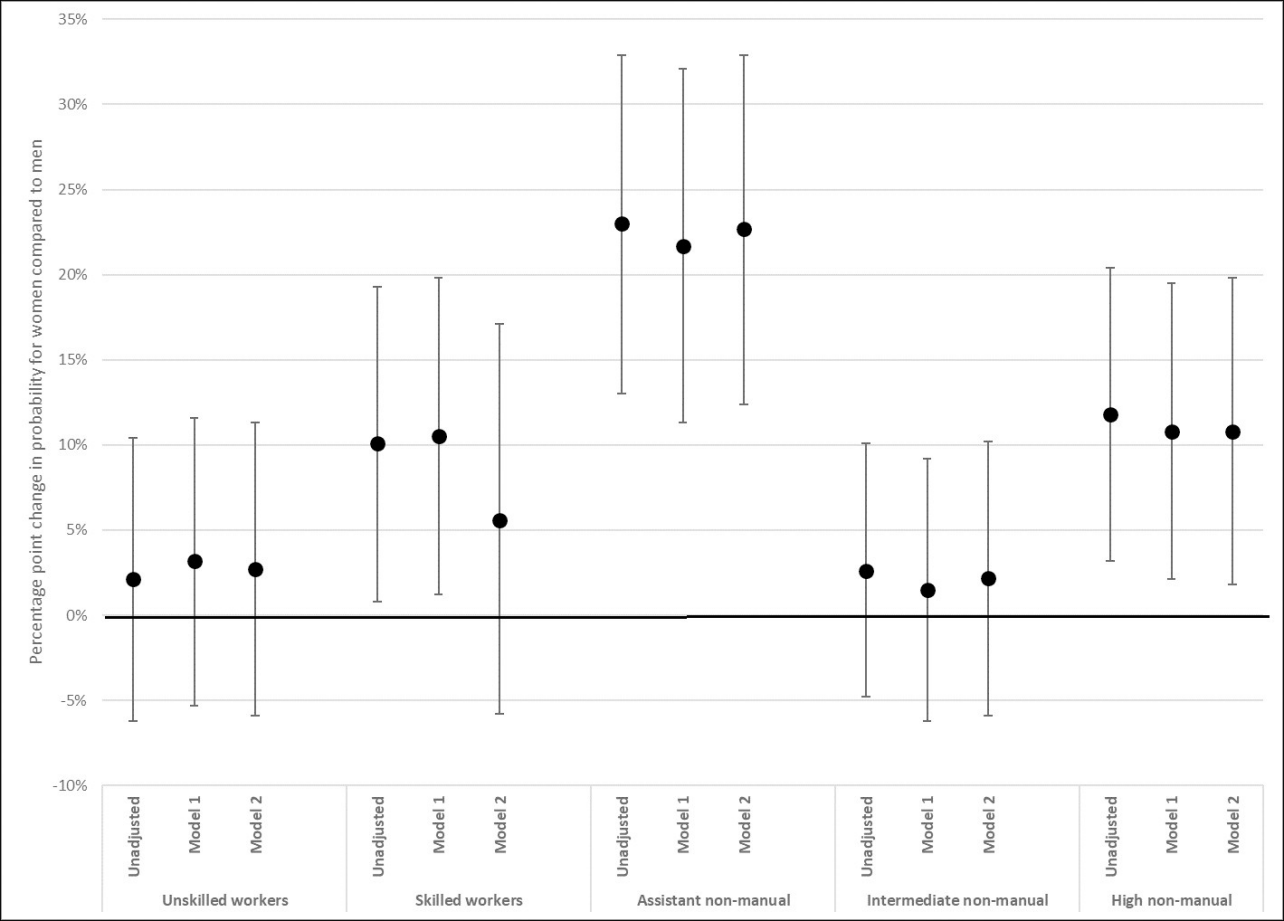
Gender differences in probability of musculoskeletal pain, by class. Coefficients from class-separate LPM. Coefficients (for woman = 1) interpreted as percentage point change in probability for musculoskeletal pain. From class-separate linear probability models, with 95% confidence intervals (Table G in S4 Appendix). Unadjusted model regresses musculoskeletal pain on gender, separately by class. Stepwise adjustment for physical working conditions (Model 1) and psychosocial working conditions (Model 2). Both unadjusted and adjusted models are controlled for age and age^2.

The inclusion of working conditions in the class separate analyses further show that the gender gap in musculoskeletal pain among assistant non-manual employees is not greatly attributed to different working conditions for men and women (Fig 6). For skilled workers, however, the pattern when adjusting for psychosocial working conditions is similar to that observed above for SRH. The percentage point increase in probability for skilled working-class women is reduced by half—indicating that psychosocial working conditions contribute also to gender differences in musculoskeletal pain within the skilled working class.

### Class-specific gender gaps in psychiatric distress

**Fig 7:**
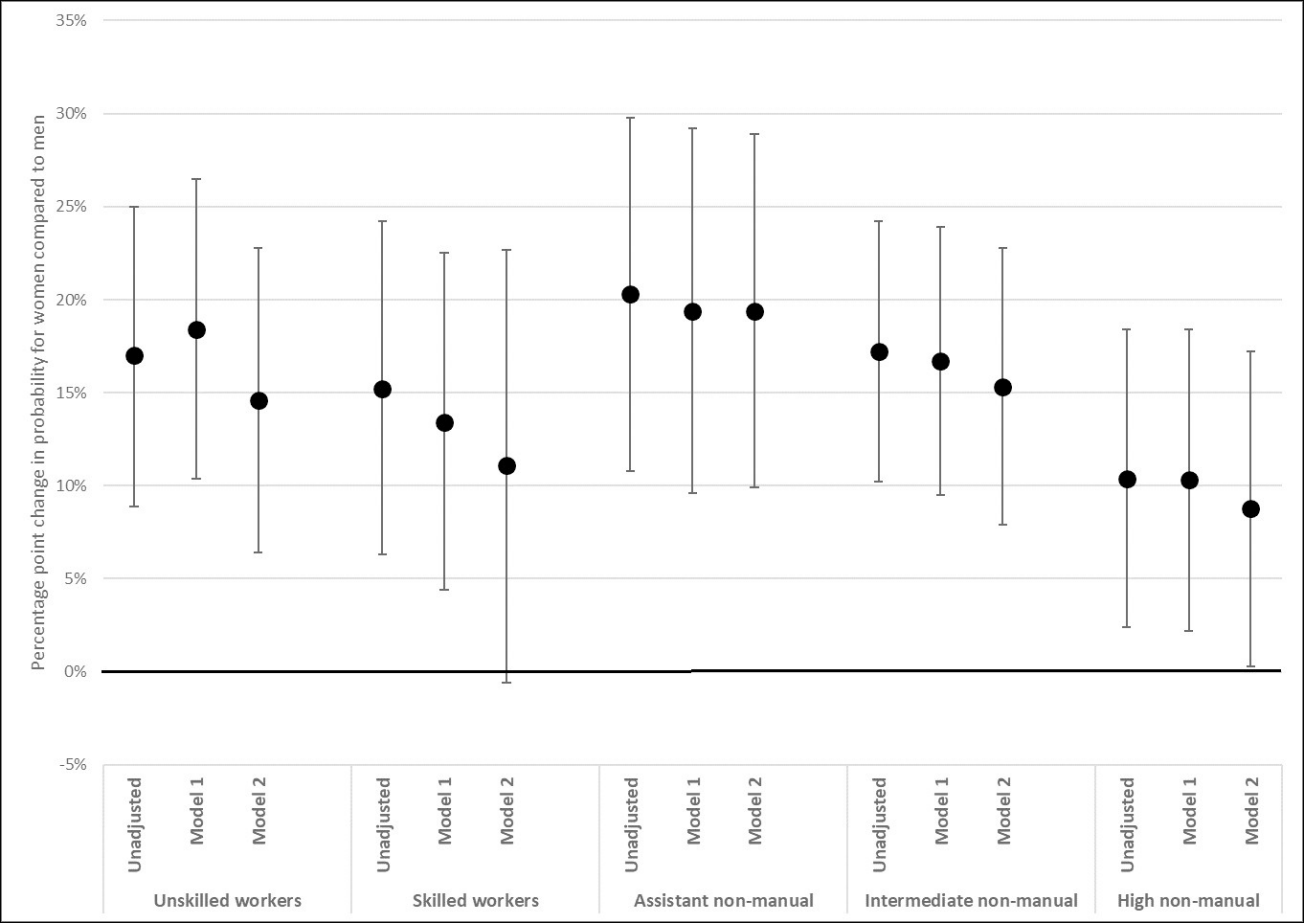
Gender differences in probability of psychiatric distress, by class. Coefficients from class-separate LPM. Coefficients (for woman = 1) interpreted as percentage point change in probability for psychiatric distress. From class-separate linear probability models, with 95% confidence intervals (Table H in S4 Appendix). Unadjusted model regresses psychiatric distress on gender, separately by class. Stepwise adjustment for physical working conditions (Model 1) and psychosocial working conditions (Model 2). Both unadjusted and adjusted models are controlled for age and age^2.

As mentioned above, the sizes of the relative gender differences in psychiatric distress are of the same magnitude for all classes, except for a narrower gender gap among high-level non-manual employees (Table A in S2 Appendix). The linear probability model, also, show no class differences in the experience of psychiatric distress (Table E in S3 Appendix). For the exploratory purposes of the study, the same class separate models as for SRH and musculoskeletal pain are nevertheless estimated also for this health outcome (Fig 7). The results show tendencies for psychosocial working conditions to attenuate the gender gaps in distress, particularly within the working classes. The confidence intervals are, however, wide and overlap substantially both between classes and between the step-wise models within classes.

## Discussion

This article set out to explore the socioeconomic variation in gender health inequality. Previous research has been mostly invested in the gender variation in socioeconomic health inequality, and larger inequality among men than among women is often described. However, different sizes of the within-gender variations in health does not tell the whole story of how between-gender differences in health arise. Given the combination of both vertical and horizontal gender segregation, as e.g. on the labour market, the drivers of gender differences in health may vary depending on the socioeconomic context. The working conditions of men and women within different classes have here been investigated as potential mechanisms behind gender differences in SRH, musculoskeletal pain and psychological distress.

As the descriptive results show, male working class and assistant non-manual employees often have more physically challenging jobs than women in their respective classes, while female intermediate and higher non-manual employees have such conditions to a large extent compared to men within their classes. Many types of physical labour is connected to physical strength, which, in turn, is conceptually connected to masculinity [32,33]. However, at the top of the vertical occupational hierarchy, physical work seems to change character from a masculine to a feminine trait. This can be connected to the human-interaction character of female work where e.g., caring also involves physical tasks [15]. Psychosocially demanding work, on the other hand, is more generally female, even though the association is particularly large among skilled workers. It can also be noted that women in general, within all classes, have a larger total burden of the demanding working conditions measured here.

For the health outcomes there are some class-specific differences between men and women for SRH and for pain. Working class women display high probability of both SRH and musculoskeletal pain. However, while working class men also have a high probability of musculoskeletal pain, they do not have a correspondingly high probability of less than good SRH. This, thus, results in smaller gender gaps in musculoskeletal pain but larger gender gaps in SRH, among the working classes compared to among non-manual employees. Assistant non-manual employees, on the other hand, display the largest overall gender gap in musculoskeletal pain, where the probability for women is more than 20 percent larger than that of men. These women, however, do not have a higher probability of pain than working class women, but men in this class have substantially lower probability. Skilled workers and higher non-manuals also display gender gaps in musculoskeletal pain, and, although not as large as among assistant non-manual employees, these differences instead stem from higher probabilities of pain among women compared to among men.

Given that sample sizes become small in the class separate analyses, interpretations regarding the contribution of working conditions are tentative. However, given the exploratory aim of the study, there are some indications of moderation by working conditions that should be noted. The gender difference in health among skilled workers can partly be attributed to psychosocial working conditions. The inclusion of these conditions explained approximately half of the gender gaps in both SRH and musculoskeletal pain for the skilled workers in this data material. These type of work demands are also particularly prominent among women in this class, both in comparison with male skilled workers, and with women in other classes.

Despite the large gender gap in SRH among unskilled workers, working conditions do not, however, seem to explain the gender gap in health among unskilled workers. Like for skilled workers, psychosocial working conditions slightly decreases the gender gap in less than good SRH also among unskilled workers, but not to the same extent. Unskilled working-class women are, also, not as subjected to these working conditions as women in skilled working class, while the opposite is true regarding skilled and unskilled working-class men.

Physical working conditions, on the other hand, is connected to small increases in the gender gaps for both SRH and musculoskeletal pain among the unskilled and skilled working classes. Increasing coefficient sizes can be a sign of interaction effects that could, for instance, indicate a higher vulnerability among working class women, compared to among working class men, for physically demanding conditions. However, given the available sample size, this this cannot be further explored in this study.

Among both assistant and higher non-manuals, the gender gaps in musculoskeletal pain are slightly attenuated by physically demanding conditions. In these classes, physical demand is also more common among women relative to men, compared to in the working class.

The case for SRH among intermediate non-manual employees is, furthermore, a notable exception where the gender gap is reversed, to men’s disadvantage, when adjusting for psychosocial working conditions. The gender difference in the reporting of these conditions among intermediate non-manual employees are still to women’s disadvantage and this could be a sign of interaction effects between psychosocial working conditions and gender, and indicate a higher vulnerability, among these non-manually employed men, for psychosocially demanding conditions. However, to further investigate this potential variation in vulnerability between men and women would call for additional studies based on a larger sample of non-manual employees.

Lastly, the results show a generally higher probability to experience distress for women than for men and no class differences are found in this regard, which is in line with previous findings [46]. The distribution table showed smaller levels of distress among female higher non-manuals compared to among other women, but this was not reflected in the analysis. However, since higher non-manual men do not experience distress more often than other men do, there could be a cause for further investigation of the vertical dimension of social conditions behind female psychiatric ill health.

### Limitations

This study is, of course, not without its limitations. Despite that the Level-of-Living survey is a large and nationally representative survey, the groups compared (by gender and class) become small, which has consequences for the statistical power to detect class-specific gender gaps and their mechanisms. Therefore, there is an increased risk of type II error, which could imply, e.g., that there may be even further variation between the classes when it comes to gender differences in health or that working conditions that are relevant for gender health differences are not identified in the class separate analyses [50]. However, if we want to explore whether the experience of working conditions matter for gender health differences, self-assessed information on working conditions are available in surveys and rarely elsewhere. Furthermore, to investigate gender differences by class, this information is needed for both men and women in a variety of occupational positions (rather than e.g. men and women at a particular workplace). There is limited available survey data material with such information. A strength of this study, thus, in relation to the aim of class-specific gender differences, is that the Level-of-Living survey provide opportunity to investigate working conditions in relation to class commonalities, not merely in relation to working conditions at, e.g., a particular workplace or in a particular profession. However, the explorative aim of the study should be emphasized and results should be viewed as a restrictive representation of the relationships between class, gender, working conditions and health.

There is also a possibility of positive selection of healthy individuals into high-ranking positions, which also contain fewer demanding working conditions. The impact of reversed causality on social inequality in health has, however, been found to be small [51]. Selection of less healthy individuals into occupations with e.g. physically demanding work has also been argued as less plausible [52]. Furthermore, health selection has been shown to be stronger *in to* work for men but *out of* work for women [53]. Women also have higher levels of sickness absence from work than men do [43,54], while higher levels of presenteeism among men has been suggested [55]. If unhealthy women are more prone to leave the work force than unhealthy men are, this could imply a possible underestimation of work-related illness among women in this sample of employees. However, these potential gender differences in health selection, and their between-class variation, cannot be ascertained here.

Women further work part time to a larger extent than men, which may have implications for gender differences in health. Part time work is more common within the working class, and in Sweden this class difference is larger for women than for men [56]. Part time employment can be viewed as a working condition and could as such have been included in the study. However, it is also a time boundary for amount of exposure to other working conditions. As a robustness check, model 4 has been estimated including also part-time employment (< = 35 hours/week) which did not alter the results.

A final issue that warrants some reflection, is the practice of using an empirical measure of *sex* as a variable for gender. Respondents in the Level-of-Living survey are identified as men or women by means of information contained in the Swedish personal identification numbers (*personnummer*) that are issued by the tax authorities to all individuals that are registered in the Swedish population (typically at birth or immigration) [57], thus based on a biological distinction. Thus, health differences between men and women in the context of this study are discussed as gender differences but their distribution over the men and women in the data material is measured by a variable that is based on a biological definition. The term “gender gap” is regularly used in reference to male-female differences in various areas [e.g. 58–61] and can be seen as a well-recognized term. Generally, in this literature, the empirical measurements are, in one way or another, biologically based (i.e. from registers or other official information). However, there are also authors that make the opposite choice, and prefer the term “sex” even when referring to social differences between men and women [14]. To disentangle sex and gender is not in focus for this study, however, whether to discuss differences between men and women in terms of sex or in terms of gender is of consequence for what interpretations that are drawn. With gender gaps in health, I refer to differences that can occur due to factors related to how the categories of men/masculinity and women/femininity are perceived and divided in society. The gender segregated labour market and gendered differences in working conditions are such factors.

## Conclusions

One conclusion that can be drawn from this study is that the difference between men’s health and women’s health is not uniform; it can vary depending on occupational class. Although class is not a large explanatory factor for the *general* female-male disadvantage in SRH, musculoskeletal pain or psychiatric distress, horizontal gender segregation of the labour market is an important aspect for understanding gender differences in health within specific occupational classes. An underlying assumption of class theory is that of shared conditions within classes and differences in conditions between classes. However, due to horizontal gender segregation, men and women have different working conditions also within classes. The present study indicate that work demand can be differently related to gender gaps in health in different classes. Psychosocially demanding conditions, e.g., contributes particularly to the gender gap in health among skilled worker, a class where these conditions are very common among women. Given the exploratory nature of this study, results are tentative and calls for further and more detailed future studies.

In order to target causes of gender health inequality, it is important to take into account not only what class means for women as well as for men, but also what gender means within specific classes–i.e. the conditions, resources and constraints for men and women within a particular group–and how this can be related to health.

## Supporting information

S1 Appendix Fig A. (TIF)

S2 Appendix Table A, Table B. (DOCX)

S3 Appendix Table C, Table D, Table E. (DOCX)

S4 Appendix Table F, Table G, Table H. (DOCX)
