# Supplementary figures and images for: Do working conditions contribute differently to gender gaps in self-rated health within different occupational classes? Evidence from the Swedish Level of Living Survey
Source: PLoS One. 2021 Jun 15;16(6):e0253119. doi: 10.1371/journal.pone.0253119 (PMC8205134; doi:10.1371/journal.pone.0253119)

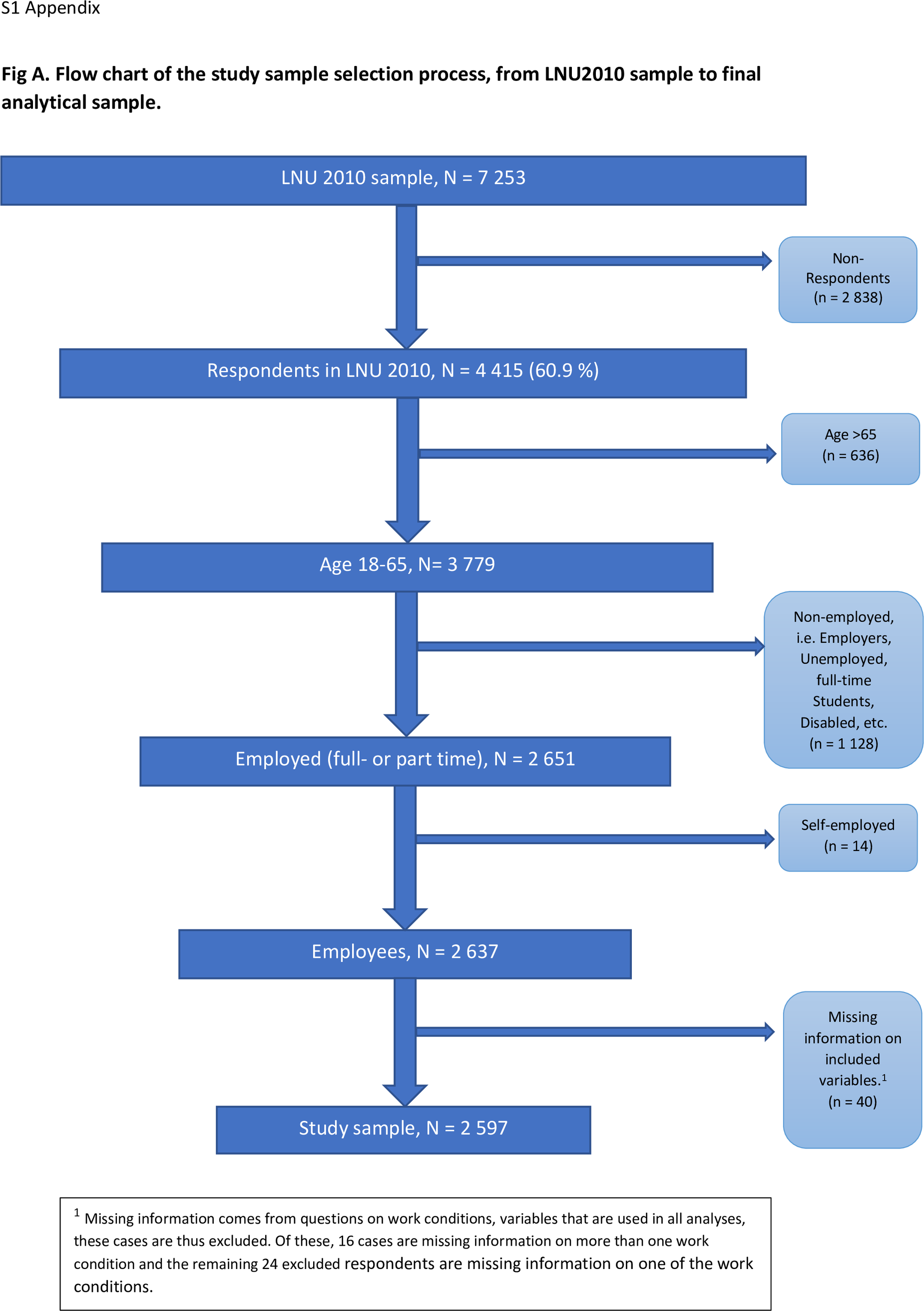

Supplement: S1 Appendix — (TIF) [file pone.0253119.s001.tif]
