# Supplementary material for: Do working conditions contribute differently to gender gaps in self-rated health within different occupational classes? Evidence from the Swedish Level of Living Survey
Source: PLoS One. 2021 Jun 15;16(6):e0253119. doi: 10.1371/journal.pone.0253119 (PMC8205134; doi:10.1371/journal.pone.0253119)
Supplement: S2 Appendix — (DOCX) [file pone.0253119.s002.docx]

**Table A. Variable distribution by gender and class, ages 18-65**

|  | **Women** | | | | | |  | **Men** | | | | | |
| --- | --- | --- | --- | --- | --- | --- | --- | --- | --- | --- | --- | --- | --- |
|  | Unskilled workers | Skilled workers | Assistant non-manual | Intermed. non-manual | Higer non-manual | **All** |  | Unskilled workers | Skilled workers | Assistant non-manual | Intermed. non-manual | Higer non-manual | **All** |
| **Physical conditions, %** |  |  |  |  |  |  |  |  |  |  |  |  |  |
| Heavy lifting | 13.7 | 25.3 | 3.0 | 5.2 | 0.9 | **8.6** |  | 32.2 | 39.5 | 17.2 | 7.0 | 1.0 | **19.0** |
| Otherwise demanding | 73.7 | 81.6 | 26.6 | 40.2 | 12.9 | **45.5** |  | 67.8 | 72.8 | 30.6 | 18.6 | 11.8 | **40.3** |
| Sweating | 32.1 | 33.9 | 3.4 | 8.2 | 2.7 | **14.8** |  | 38.8 | 40.2 | 12.1 | 6.1 | 2.1 | **20.0** |
| Repetetive movements | 59.9 | 48.9 | 63.1 | 33.2 | 42.2 | **48.0** |  | 59.4 | 55.8 | 43.9 | 42.4 | 37.8 | **48.0** |
| Unsuitable positions | 58.0 | 74.7 | 18.0 | 31.5 | 9.3 | **36.5** |  | 58.0 | 69.6 | 29.3 | 21.0 | 6.6 | **36.9** |
| Sitting down | 14.1 | 5.2 | 62.2 | 39.4 | 69.3 | **39.0** |  | 27.6 | 15.6 | 52.2 | 64.0 | 79.9 | **48.2** |
| **Psychosocial conditions, %** |  |  |  |  |  |  |  |  |  |  |  |  |  |
| High demands/low control | 26.7 | 47.1 | 31.8 | 38.0 | 22.7 | **33.0** |  | 24.1 | 16.3 | 27.4 | 19.8 | 18.1 | **20.5** |
| Emotional work | 22.5 | 49.4 | 18.9 | 40.2 | 31.6 | **32.3** |  | 8.4 | 6.5 | 9.6 | 11.6 | 9.7 | **9.2** |
| Non-flexible hours | 69.8 | 69.5 | 31.8 | 35.6 | 12.9 | **42.6** |  | 66.1 | 52.9 | 31.8 | 17.7 | 10.1 | **35.4** |
| No errands | 70.6 | 67.2 | 43.3 | 36.7 | 14.2 | **45.2** |  | 60.5 | 39.9 | 32.5 | 18.0 | 7.3 | **31.0** |
| Lacking social support | 11.8 | 1.7 | 10.3 | 6.0 | 7.1 | **7.6** |  | 10.5 | 5.8 | 8.3 | 5.5 | 5.2 | **6.9** |
| **Health, %** |  |  |  |  |  |  |  |  |  |  |  |  |  |
| Less than good SRH | 27.9 | 29.3 | 21.9 | 19.3 | 13.8 | **21.9** |  | 14.3 | 18.1 | 14.6 | 18.6 | 11.8 | **15.7** |
| *Relative difference (w/m)* | *1.95* | *1.62* | *1.50* | *1.04* | *1.17* | *1.39* |  |  |  |  |  |  |  |
| Musculoskeletal pain | 53.1 | 62.1 | 58.8 | 50.3 | 48.0 | **53.6** |  | 52.8 | 50.0 | 33.1 | 47.6 | 36.1 | **45.0** |
| *Relative difference (w/m)* | *1.01* | *1.24* | *1.78* | *1.06* | *1.33* | *1.19* |  |  |  |  |  |  |  |
| Psychiatric distress | 43.9 | 40.2 | 45.1 | 44.0 | 35.6 | **42.2** |  | 26.2 | 24.6 | 26.8 | 26.8 | 24.3 | **25.7** |
| *Relative difference (w/m)* | *1.68* | *1.63* | *1.68* | *1.64* | *1.47* | *1.64* |  |  |  |  |  |  |  |
| **n** | **262.0** | **174.0** | **233.0** | **368.0** | **225.0** | **1262.0** |  | **286.0** | **276.0** | **157.0** | **328.0** | **288.0** | **1335.0** |
| *%* | *20.8* | *13.8* | *18.5* | *29.2* | *17.8* | *100.0* |  | *21.4* | *20.7* | *11.8* | *24.6* | *21.6* | *100.0* |

| **Table B. Five most common occupational groups within classes, by sex. (4-digit ISCO88)^1^** | | | | | | |
| --- | --- | --- | --- | --- | --- | --- |
|  | **MEN** | | | **WOMEN** | | |
| **Class** | **ISCO88** | **Occupational category** | **%** | **ISCO88** | **Occupational category** | **%** |
| Unskilled workers | 8324 | Heavy truck and lorry drivers | 9.39 | 5133 | Home-based personal care workers | 24.50 |
|  | 5220 | Shop, stall and market salespersons and demonstrators | 8.66 | 9132 | Helpers and cleaners in offices, hotels and other establishments | 20.48 |
|  | 4131 | Stock clerks | 7.22 | 5220 | Shop, stall and market salespersons and demonstrators | 18.88 |
|  | 8334 | Lifting-truck operators | 5.78 | 5132 | Institution-based personal care workers | 4.82 |
|  | 9132 | Helpers and cleaners in offices, hotels and other establishments | 4.69 | 4211 | Cashiers and ticket clerks | 4.02 |
| Skilled workers | 7124 | Carpenters and joiners | 9.49 | 5132 | Institution-based personal care workers | 56.40 |
|  | 7137 | Building and related electricians | 8.76 | 5131 | Child-care workers | 17.44 |
|  | 8211 | Machine-tool operators | 5.11 | 5122 | Cooks | 6.40 |
|  | 7233 | Agricultural- or industrial-machinery mechanics and fitters | 5.11 | 5123 | Waiters, waitresses and bartenders | 2.91 |
|  | 5132 | Institution-based personal care workers | 5.11 | 5141 | Hairdressers, barbers, beauticians and related workers | 1.74 |
| Assistant non-manual | 3415 | Technical and commercial sales representatives | 25.64 | 4190 | Other office clerks | 16.09 |
|  | 4190 | Other office clerks | 7.05 | 3431 | Administrative secretaries and related associate professionals | 10.87 |
|  | 3450 | Police inspectors and detectives | 6.41 | 4121 | Accounting and bookkeeping clerks | 9.57 |
|  | 1314 | Managers of small enterprises in wholesale and retail trade | 5.13 | 3415 | Technical and commercial sales representatives | 9.57 |
|  | 3431 | Administrative secretaries and related associate professionals | 4.49 | 5220 | Shop, stall and market salespersons and demonstrators | 6.52 |

| Intermed. non-manual | 3115 | Mechanical engineering technicians | 8.00 | 3320 | Pre-primary education teaching associate professionals | 16.89 |
| --- | --- | --- | --- | --- | --- | --- |
|  | 2145 | Mechanical engineers | 6.77 | 3231 | Nursing associate professionals | 11.44 |
|  | 2331 | Primary education teaching professionals | 6.15 | 2331 | Primary education teaching professionals | 11.17 |
|  | 3112 | Civil engineering technicians | 5.85 | 2419 | Business professionals not elsewhere classified | 7.08 |
|  | 3121 | Computer assistants | 5.23 | 2230 | Nursing and midwifery professionals | 4.63 |
| Higher non-manual | 2139 | Computing professionals not elsewhere classified | 9.86 | 1229 | Production and operations managers not elsewhere classified | 9.63 |
|  | 1210 | Directors and chief executives | 9.51 | 2446 | Social work professionals | 9.17 |
|  | 2131 | Computer systems designers, analysts and programmers | 8.45 | 2320 | Secondary education teaching professionals | 7.34 |
|  | 2221 | Medical doctors | 5.99 | 2310 | College, university and higher education teaching professionals | 6.88 |
|  | 2419 | Business professionals not elsewhere classified | 5.63 | 2411 | Accountants | 5.50 |

**^1^ Note:** The same 4-digit ISCO88 can appear in more than one class, but correspond to different occupations. For example ISCO88-code 5132 is found in the top 5 for both skilled and unskilled working class women. For unskilled workers this corresponds to various caretakers or assistants in hospitals with no or low skill requirements, while for skilled workers ISCO88-code 5132 refers to assistant nurses.
