# Supplementary material for: Do working conditions contribute differently to gender gaps in self-rated health within different occupational classes? Evidence from the Swedish Level of Living Survey
Source: PLoS One. 2021 Jun 15;16(6):e0253119. doi: 10.1371/journal.pone.0253119 (PMC8205134; doi:10.1371/journal.pone.0253119)
Supplement: S3 Appendix — (DOCX) [file pone.0253119.s003.docx]

**Table C. Probability of less than good SRH, by gender and class. LPM, N=2 597**

|  | Unadjusted | | Model 1 | | Model 2 | |
| --- | --- | --- | --- | --- | --- | --- |
| **Self-rated health** | coef. | 95% CI | coef. | 95% CI | coef. | 95% CI |
| Woman | 0.062 | 0.032 - 0.092 | 0.063 | 0.033 - 0.093 | 0.023 | -0.036 - 0.081 |
| **Class** |  |  |  |  |  |  |
| Unskilled worker |  |  | 0.086 | 0.040 - 0.133 | 0.033 | -0.023 - 0.090 |
| Skilled worker |  |  | 0.105 | 0.056 - 0.153 | 0.07 | 0.010 - 0.130 |
| Assistant non-manual |  |  | 0.056 | 0.007 - 0.105 | 0.039 | -0.029 - 0.107 |
| Interm. non-manual |  |  | 0.059 | 0.018 - 0.101 | 0.072 | 0.016 - 0.128 |
| Higher non-manual |  |  | Ref |  | Ref |  |
| **Woman*Class** |  |  |  |  |  |  |
| W*Unskilled worker |  |  |  |  | 0.116 | 0.026 - 0.205 |
| W*Skilled worker |  |  |  |  | 0.085 | -0.015 - 0.186 |
| W*Assistant non-manual |  |  |  |  | 0.04 | -0.057 - 0.137 |
| W*Interm. non-manual |  |  |  |  | -0.017 | -0.100 - 0.066 |
| W*Higher non-manual |  |  |  |  | Ref |  |
| Constant | 0.219 | 0.055 - 0.384 | 0.085 | -0.094 - 0.264 | 0.092 | -0.089 - 0.273 |
| R-squared | 0.011 |  | 0.018 |  | 0.023 |  |
| CI=Confidence interval | | All models controlled for age and age^2 | | | | |

**Table D. Probability of musculoskeletal pain, by gender and class. LPM, N=2 597**

|  | Unadjusted | | Model 1 | | Model 2 | |
| --- | --- | --- | --- | --- | --- | --- |
| **Musculoskeletal pain** | coef. | 95% CI | coef. | 95% CI | coef. | 95% CI |
| Woman | 0.084 | 0.046 / 0.122 | 0.087 | 0.049 / 0.126 | 0.12 | 0.034 / 0.206 |
| **Class** |  |  |  |  |  |  |
| Unskilled worker |  |  | 0.154 | 0.093 / 0.214 | 0.197 | 0.117 / 0.278 |
| Skilled worker |  |  | 0.166 | 0.104 / 0.228 | 0.171 | 0.090 / 0.253 |
| Assistant non-manual |  |  | 0.084 | 0.019 / 0.149 | 0.011 | -0.083 / 0.105 |
| Interm. non-manual |  |  | 0.076 | 0.019 / 0.133 | 0.122 | 0.044 / 0.200 |
| Higher non-manual |  |  | Ref |  | Ref |  |
| **Woman*Class** |  |  |  |  |  |  |
| W*Unskilled worker |  |  |  |  | -0.100 | -0.219 / 0.020 |
| W*Skilled worker |  |  |  |  | -0.014 | -0.139 / 0.112 |
| W*Assistant non-manual |  |  |  |  | 0.110 | -0.019 / 0.240 |
| W*Interm. non-manual |  |  |  |  | -0.093 | -0.207 / 0.021 |
| W*Higher non-manual |  |  |  |  | Ref |  |
| Constant | 0.052 | -0.159 / 0.263 | -0.182 | -0.409 / 0.044 | -0.161 | -0.390 / 0.068 |
| R-squared | 0.019 |  | 0.032 |  | 0.037 |  |
| CI=Confidence interval | | All models controlled for age and age^2 | | | | |

**Table E. Probability of psychiatric distress, by gender and class. LPM, N=2 597**

|  | Unadjusted | | Model 1 | | Model 2 | |
| --- | --- | --- | --- | --- | --- | --- |
| **Psychiatric distress** | coef. | 95% CI | coef. | 95% CI | coef. | 95% CI |
| Woman | 0.166 | 0.130 / 0.201 | 0.161 | 0.125 / 0.197 | 0.11 | 0.030 / 0.190 |
| **Class** |  |  |  |  |  |  |
| Unskilled worker |  |  | 0.04 | -0.016 / 0.097 | 0.012 | -0.059 / 0.083 |
| Skilled worker |  |  | 0.019 | -0.040 / 0.077 | -0.003 | -0.075 / 0.069 |
| Assistant non-manual |  |  | 0.055 | -0.007 / 0.117 | 0.015 | -0.071 / 0.100 |
| Interm. non-manual |  |  | 0.05 | -0.002 / 0.103 | 0.022 | -0.047 / 0.091 |
| Higher non-manual |  |  | Ref |  | Ref |  |
| **Woman*Class** |  |  |  |  |  |  |
| W*Unskilled worker |  |  |  |  | 0.064 | -0.049 / 0.176 |
| W*Skilled worker |  |  |  |  | 0.049 | -0.071 / 0.169 |
| W*Assistant non-manual |  |  |  |  | 0.081 | -0.042 / 0.205 |
| W*Interm. non-manual |  |  |  |  | 0.063 | -0.044 / 0.169 |
| W*Higher non-manual |  |  |  |  | Ref |  |
| Constant | 0.308 | 0.105 / 0.510 | 0.26 | 0.045 / 0.474 | 0.28 | 0.063 / 0.497 |
| R-squared | 0.033 |  | 0.034 |  | 0.035 |  |
| CI=Confidence interval | | All models controlled for age and age^2 | | | | |
