# Supplementary material for: Do working conditions contribute differently to gender gaps in self-rated health within different occupational classes? Evidence from the Swedish Level of Living Survey
Source: PLoS One. 2021 Jun 15;16(6):e0253119. doi: 10.1371/journal.pone.0253119 (PMC8205134; doi:10.1371/journal.pone.0253119)
Supplement: S4 Appendix — (DOCX) [file pone.0253119.s004.docx]

**Table F. Probability for less than good SRH, for men and women. Models estimated separately by class, LPM.**

|  | **Panel i: Unskilled workers** | | | | | |
| --- | --- | --- | --- | --- | --- | --- |
|  | Unadjusted | | Model 1 | | Model 2 | |
|  | coef. | 95% CI | coef. | 95% CI | coef. | 95% CI |
| Woman | 0.148 | 0.080 / 0.217 | 0.155 | 0.084 / 0.226 | 0.137 | 0.067 / 0.207 |
| Physically strenuous |  |  | 0.027 | -0.002 / 0.056 |  |  |
| Repetitive |  |  | 0.052 | -0.018 / 0.122 |  |  |
| Sitting |  |  | 0.005 | -0.081 / 0.090 |  |  |
| Demand/Control |  |  |  |  | 0.098 | 0.012 / 0.185 |
| Emotional |  |  |  |  | 0.046 | -0.058 / 0.150 |
| Non-flexible |  |  |  |  | -0.001 | -0.081 / 0.079 |
| No errands |  |  |  |  | 0.02 | -0.058 / 0.098 |
| Lacking support |  |  |  |  | 0.043 | -0.065 / 0.152 |
| Constant | -0.296 | -0.591 / -0.001 | -0.361 | -0.665 / -0.057 | -0.321 | -0.638 / -0.004 |
| Observations | 548 |  | 548 |  | 548 |  |
| R-squared | 0.043 |  | 0.055 |  | 0.059 |  |
|  | **Panel ii: Skilled workers** | | | | | |
|  | Unadjusted | | Model 1 | | Model 2 | |
|  | coef. | 95% CI | coef. | 95% CI | coef. | 95% CI |
| Woman | 0.110 | 0.028 / 0.192 | 0.115 | 0.031 / 0.198 | 0.064 | -0.043 / 0.170 |
| Physically strenuous |  |  | 0.013 | -0.020 / 0.045 |  |  |
| Repetitive |  |  | 0.144 | 0.068 / 0.220 |  |  |
| Sitting |  |  | -0.049 | -0.164 / 0.067 |  |  |
| Demand/Control |  |  |  |  | -0.006 | -0.102 / 0.090 |
| Emotional |  |  |  |  | 0.075 | -0.043 / 0.194 |
| Non-flexible |  |  |  |  | 0.042 | -0.039 / 0.123 |
| No errands |  |  |  |  | 0.035 | -0.051 / 0.121 |
| Lacking support |  |  |  |  | 0.022 | -0.174 / 0.218 |
| Constant | 0.264 | -0.122 / 0.650 | 0.122 | -0.296 / 0.539 | 0.215 | -0.165 / 0.595 |
| Observations | 450 |  | 450 |  | 450 |  |
| R-squared | 0.021 |  | 0.054 |  | 0.03 |  |
| CI=Confidence Interval | | All models controlled for age and age^2 | | |  |  |

**(…cont) Table F. Probability for less than good SRH, for men and women. Models estimated separately by**

**class, LPM.**

|  | **Panel iii: Assistant non-manual** | | | | | |
| --- | --- | --- | --- | --- | --- | --- |
|  | Unadjusted | | Model 1 | | Model 2 | |
|  | coef. | 95% CI | coef. | 95% CI | coef. | 95% CI |
| Woman | 0.062 | -0.015 / 0.139 | 0.063 | -0.017 / 0.142 | 0.047 | -0.029 / 0.123 |
| Physically strenuous |  |  | 0.024 | -0.017 / 0.064 |  |  |
| Repetitive |  |  | 0.037 | -0.045 / 0.119 |  |  |
| Sitting |  |  | -0.007 | -0.093 / 0.078 |  |  |
| Demand/Control |  |  |  |  | 0.055 | -0.033 / 0.143 |
| Emotional |  |  |  |  | 0.105 | -0.020 / 0.231 |
| Non-flexible |  |  |  |  | 0.01 | -0.089 / 0.109 |
| No errands |  |  |  |  | 0.005 | -0.087 / 0.098 |
| Lacking support |  |  |  |  | 0.165 | 0.003 / 0.327 |
| Constant | 0.225 | -0.170 / 0.620 | 0.147 | -0.284 / 0.578 | 0.184 | -0.233 / 0.601 |
| Observations | 390 |  | 390 |  | 390 |  |
| R-squared | 0.017 |  | 0.023 |  | 0.047 |  |
|  | **Panel iv: Intermediate non-manual** | | | | | |
|  | Unadjusted | | Model 1 | | Model 2 | |
|  | coef. | 95% CI | coef. | 95% CI | coef. | 95% CI |
| Woman | 0.004 | -0.054 / 0.062 | -0.009 | -0.068 / 0.051 | -0.043 | -0.104 / 0.019 |
| Physically strenuous |  |  | 0.032 | -0.002 / 0.067 |  |  |
| Repetitive |  |  | -0.024 | -0.090 / 0.042 |  |  |
| Sitting |  |  | 0 | -0.076 / 0.076 |  |  |
| Demand/Control |  |  |  |  | 0.058 | -0.015 / 0.132 |
| Emotional |  |  |  |  | 0.078 | 0.003 / 0.153 |
| Non-flexible |  |  |  |  | 0.001 | -0.076 / 0.079 |
| No errands |  |  |  |  | 0.067 | -0.006 / 0.140 |
| Lacking support |  |  |  |  | 0.134 | -0.016 / 0.283 |
| Constant | 0.578 | 0.129 / 1.028 | 0.587 | 0.143 / 1.030 | 0.56 | 0.113 / 1.006 |
| Observations | 696 |  | 696 |  | 696 |  |
| R-squared | 0.014 |  | 0.023 |  | 0.044 |  |
| CI=Confidence Interval | | All models controlled for age and age^2 | | |  |  |

**(…cont) Table F. Probability for less than good SRH, for men and women. Models estimated separately by**

**class, LPM.**

|  | **Panel v: Higher non-manual** | | | | | |
| --- | --- | --- | --- | --- | --- | --- |
|  | Unadjusted | | Model 1 | | Model 2 | |
|  | coef. | 95% CI | coef. | 95% CI | coef. | 95% CI |
| Woman | 0.022 | -0.036 / 0.081 | 0.018 | -0.040 / 0.076 | 0.014 | -0.047 / 0.075 |
| Physically strenuous |  |  | 0.025 | -0.026 / 0.076 |  |  |
| Repetitive |  |  | 0.053 | -0.009 / 0.115 |  |  |
| Sitting |  |  | -0.010 | -0.086 / 0.066 |  |  |
| Demand/Control |  |  |  |  | 0.045 | -0.034 / 0.124 |
| Emotional |  |  |  |  | 0.028 | -0.056 / 0.112 |
| Non-flexible |  |  |  |  | 0.076 | -0.049 / 0.200 |
| No errands |  |  |  |  | -0.053 | -0.179 / 0.072 |
| Lacking support |  |  |  |  | 0.075 | -0.069 / 0.219 |
| Constant | 0.312 | -0.177 / 0.801 | 0.261 | -0.228 / 0.751 | 0.296 | -0.195 / 0.788 |
| Observations | 513 |  | 513 |  | 513 |  |
| R-squared | 0.004 |  | 0.012 |  | 0.015 |  |
| CI=Confidence Interval | | All models controlled for age and age^2 | | |  |  |

**Table G. Probability for musculoskeletal pain, for men and women. Models estimated separately by class, LPM.**

|  | **Panel i: Unskilled workers** | | | | | |
| --- | --- | --- | --- | --- | --- | --- |
|  | Unadjusted | | Model 1 | | Model 2 | |
|  | coef. | 95% CI | coef. | 95% CI | coef. | 95% CI |
| Woman | 0.021 | -0.062 - 0.104 | 0.032 | -0.053 - 0.116 | 0.027 | -0.059 - 0.113 |
| Physically strenuous |  |  | 0.041 | 0.003 - 0.078 |  |  |
| Repetitive |  |  | 0.072 | -0.016 - 0.159 |  |  |
| Sitting |  |  | 0.012 | -0.093 - 0.116 |  |  |
| Demand/Control |  |  |  |  | 0.017 | -0.082 - 0.117 |
| Emotional |  |  |  |  | -0.073 | -0.193 - 0.047 |
| Non-flexible |  |  |  |  | -0.007 | -0.105 - 0.091 |
| No errands |  |  |  |  | 0.032 | -0.064 - 0.128 |
| Lacking support |  |  |  |  | 0.116 | -0.014 - 0.246 |
| Constant | 0.117 | -0.270 - 0.505 | 0.024 | -0.371 - 0.420 | 0.08 | -0.324 - 0.484 |
| Observations | 548 |  | 548 |  | 548 |  |
| R-squared | 0.032 |  | 0.049 |  | 0.041 |  |
|  | **Panel ii: Skilled workers** | | | | | |
|  | Unadjusted | | Model 1 | | Model 2 | |
|  | coef. | 95% CI | coef. | 95% CI | coef. | 95% CI |
| Woman | 0.101 | 0.008 - 0.193 | 0.105 | 0.012 - 0.198 | 0.056 | -0.058 - 0.171 |
| Physically strenuous |  |  | 0.037 | -0.005 - 0.079 |  |  |
| Repetitive |  |  | 0.075 | -0.017 - 0.167 |  |  |
| Sitting |  |  | -0.033 | -0.194 - 0.129 |  |  |
| Demand/Control |  |  |  |  | 0.045 | -0.065 - 0.155 |
| Emotional |  |  |  |  | 0.046 | -0.076 - 0.169 |
| Non-flexible |  |  |  |  | 0.009 | -0.088 - 0.106 |
| No errands |  |  |  |  | 0.05 | -0.049 - 0.148 |
| Lacking support |  |  |  |  | 0.12 | -0.106 - 0.345 |
| Constant | -0.19 | -0.636 - 0.256 | -0.346 | -0.811 - 0.119 | -0.219 | -0.674 - 0.236 |
| Observations | 450 |  | 450 |  | 450 |  |
| R-squared | 0.052 |  | 0.069 |  | 0.061 |  |
| CI=Confidence Interval | | All models controlled for age and age^2 | | |  |  |

**(…cont) Table G. Probability for musculoskeletal pain, for men and women. Models estimated separately by**

**class, LPM.**

|  | **Panel iii: Assistant non-manual** | | | | | |
| --- | --- | --- | --- | --- | --- | --- |
|  | Unadjusted | | Model 1 | | Model 2 | |
|  | coef. | 95% CI | coef. | 95% CI | coef. | 95% CI |
| Woman | 0.230 | 0.130 - 0.329 | 0.217 | 0.113 - 0.321 | 0.227 | 0.124 - 0.329 |
| Physically strenuous |  |  | 0.006 | -0.045 - 0.057 |  |  |
| Repetitive |  |  | 0.075 | -0.027 - 0.177 |  |  |
| Sitting |  |  | -0.013 | -0.124 - 0.099 |  |  |
| Demand/Control |  |  |  |  | -0.003 | -0.116 - 0.110 |
| Emotional |  |  |  |  | -0.023 | -0.167 - 0.122 |
| Non-flexible |  |  |  |  | -0.012 | -0.131 - 0.107 |
| No errands |  |  |  |  | 0.039 | -0.072 - 0.149 |
| Lacking support |  |  |  |  | 0.113 | -0.046 - 0.272 |
| Constant | -0.264 | -0.772 - 0.245 | -0.322 | -0.850 - 0.206 | -0.293 | -0.820 - 0.234 |
| Observations | 390 |  | 390 |  | 390 |  |
| R-squared | 0.083 |  | 0.088 |  | 0.089 |  |
|  | **Panel iv: Intermediate non-manual** | | | | | |
|  | Unadjusted | | Model 1 | | Model 2 | |
|  | coef. | 95% CI | coef. | 95% CI | coef. | 95% CI |
| Woman | 0.026 | -0.048 - 0.101 | 0.015 | -0.062 - 0.092 | 0.022 | -0.059 - 0.102 |
| Physically strenuous |  |  | 0.055 | 0.016 - 0.095 |  |  |
| Repetitive |  |  | 0.119 | 0.035 - 0.203 |  |  |
| Sitting |  |  | -0.019 | -0.116 - 0.077 |  |  |
| Demand/Control |  |  |  |  | 0.093 | 0.005 - 0.182 |
| Emotional |  |  |  |  | -0.093 | -0.184 - -0.002 |
| Non-flexible |  |  |  |  | 0.039 | -0.061 - 0.138 |
| No errands |  |  |  |  | 0.041 | -0.056 - 0.137 |
| Lacking support |  |  |  |  | -0.036 | -0.193 - 0.122 |
| Constant | 0.411 | -0.163 - 0.985 | 0.279 | -0.293 - 0.851 | 0.352 | -0.222 - 0.926 |
| Observations | 696 |  | 696 |  | 696 |  |
| R-squared | 0.002 |  | 0.028 |  | 0.017 |  |
| CI=Confidence Interval | | All models controlled for age and age^2 | | |  |  |

**(…cont) Table G. Probability for musculoskeletal pain, for men and women. Models estimated separately by**

**class, LPM.**

|  | **Panel v: Higher non-manual** | | | | | |
| --- | --- | --- | --- | --- | --- | --- |
|  | Unadjusted | | Model 1 | | Model 2 | |
|  | coef. | 95% CI | coef. | 95% CI | coef. | 95% CI |
| Woman | 0.118 | 0.032 - 0.204 | 0.108 | 0.021 - 0.195 | 0.108 | 0.018 - 0.198 |
| Physically strenuous |  |  | 0.043 | -0.026 - 0.113 |  |  |
| Repetitive |  |  | 0.154 | 0.065 - 0.242 |  |  |
| Sitting |  |  | -0.017 | -0.125 - 0.091 |  |  |
| Demand/Control |  |  |  |  | 0.076 | -0.036 - 0.188 |
| Emotional |  |  |  |  | 0.072 | -0.045 - 0.189 |
| Non-flexible |  |  |  |  | -0.006 | -0.154 - 0.142 |
| No errands |  |  |  |  | -0.108 | -0.263 - 0.046 |
| Lacking support |  |  |  |  | -0.097 | -0.268 - 0.073 |
| Constant | -0.006 | -0.743 - 0.731 | -0.143 | -0.866 - 0.581 | 0.014 | -0.726 - 0.753 |
| Observations | 513 |  | 513 |  | 513 |  |
| R-squared | 0.016 |  | 0.043 |  | 0.028 |  |
| CI=Confidence Interval | | All models controlled for age and age^2 | | |  |  |

**Table H. Probability for psychiatric distress, for men and women. Models estimated separately by class, LPM.**

|  | **Panel i: Unskilled workers** | | | | | |
| --- | --- | --- | --- | --- | --- | --- |
|  | Unadjusted | | Model 1 | | Model 2 | |
|  | coef. | 95% CI | coef. | 95% CI | coef. | 95% CI |
| Woman | 0.17 | 0.089 - 0.250 | 0.184 | 0.104 - 0.265 | 0.146 | 0.064 - 0.228 |
| Physically strenuous |  |  | 0.068 | 0.035 - 0.101 |  |  |
| Repetitive |  |  | 0.076 | -0.004 - 0.157 |  |  |
| Sitting |  |  | 0 | -0.096 - 0.095 |  |  |
| Demand/Control |  |  |  |  | 0.091 | -0.008 - 0.190 |
| Emotional |  |  |  |  | 0.133 | 0.013 - 0.254 |
| Non-flexible |  |  |  |  | 0.057 | -0.035 - 0.148 |
| No errands |  |  |  |  | -0.006 | -0.097 - 0.086 |
| Lacking support |  |  |  |  | 0.085 | -0.041 - 0.211 |
| Constant | 0.458 | 0.076 - 0.841 | 0.353 | -0.029 - 0.734 | 0.395 | -0.003 - 0.792 |
| Observations | 548 |  | 548 |  | 548 |  |
| R-squared | 0.038 |  | 0.079 |  | 0.065 |  |
|  | **Panel ii: Skilled workers** | | | | | |
|  | Unadjusted | | Model 1 | | Model 2 | |
|  | coef. | 95% CI | coef. | 95% CI | coef. | 95% CI |
| Woman | 0.152 | 0.063 - 0.242 | 0.134 | 0.044 - 0.225 | 0.111 | -0.006 - 0.227 |
| Physically strenuous |  |  | -0.022 | -0.058 - 0.014 |  |  |
| Repetitive |  |  | 0.038 | -0.048 - 0.125 |  |  |
| Sitting |  |  | -0.176 | -0.296 - -0.056 |  |  |
| Demand/Control |  |  |  |  | 0.113 | 0.003 - 0.222 |
| Emotional |  |  |  |  | -0.013 | -0.145 - 0.118 |
| Non-flexible |  |  |  |  | 0.027 | -0.061 - 0.115 |
| No errands |  |  |  |  | 0.036 | -0.056 - 0.127 |
| Lacking support |  |  |  |  | 0.036 | -0.191 - 0.263 |
| Constant | 0.216 | -0.198 - 0.630 | 0.276 | -0.161 - 0.713 | 0.2 | -0.220 - 0.621 |
| Observations | 450 |  | 450 |  | 450 |  |
| R-squared | 0.03 |  | 0.042 |  | 0.045 |  |
| CI=Confidence Interval | | All models controlled for age and age^2 | | |  |  |

**(…cont) Table H. Probability for psychiatric distress, for men and women. Models estimated separately by**

**class, LPM.**

|  | **Panel iii: Assistant non-manual** | | | | | |
| --- | --- | --- | --- | --- | --- | --- |
|  | Unadjusted | | Model 1 | | Model 2 | |
|  | coef. | 95% CI | coef. | 95% CI | coef. | 95% CI |
| Woman | 0.203 | 0.108 - 0.298 | 0.194 | 0.096 - 0.292 | 0.194 | 0.099 - 0.289 |
| Physically strenuous |  |  | 0.049 | -0.001 - 0.099 |  |  |
| Repetitive |  |  | 0.094 | -0.008 - 0.197 |  |  |
| Sitting |  |  | 0.067 | -0.042 - 0.177 |  |  |
| Demand/Control |  |  |  |  | 0.119 | 0.009 - 0.230 |
| Emotional |  |  |  |  | 0.041 | -0.099 - 0.180 |
| Non-flexible |  |  |  |  | -0.041 | -0.163 - 0.080 |
| No errands |  |  |  |  | 0 | -0.113 - 0.112 |
| Lacking support |  |  |  |  | 0.227 | 0.056 - 0.399 |
| Constant | 0.328 | -0.173 - 0.829 | 0.165 | -0.349 - 0.680 | 0.362 | -0.152 - 0.876 |
| Observations | 390 |  | 390 |  | 390 |  |
| R-squared | 0.047 |  | 0.069 |  | 0.081 |  |
|  | **Panel iv: Intermediate non-manual** | | | | | |
|  | Unadjusted | | Model 1 | | Model 2 | |
|  | coef. | 95% CI | coef. | 95% CI | coef. | 95% CI |
| Woman | 0.172 | 0.102 - 0.242 | 0.167 | 0.095 - 0.239 | 0.153 | 0.079 - 0.228 |
| Physically strenuous |  |  | 0.019 | -0.019 - 0.057 |  |  |
| Repetitive |  |  | 0.099 | 0.020 - 0.179 |  |  |
| Sitting |  |  | -0.032 | -0.121 - 0.057 |  |  |
| Demand/Control |  |  |  |  | 0.053 | -0.032 - 0.138 |
| Emotional |  |  |  |  | 0.012 | -0.076 - 0.100 |
| Non-flexible |  |  |  |  | 0.01 | -0.083 - 0.103 |
| No errands |  |  |  |  | 0.017 | -0.072 - 0.107 |
| Lacking support |  |  |  |  | 0.144 | -0.012 - 0.300 |
| Constant | 0.439 | -0.105 - 0.982 | 0.359 | -0.178 - 0.896 | 0.402 | -0.136 - 0.940 |
| Observations | 696 |  | 696 |  | 696 |  |
| R-squared | 0.033 |  | 0.044 |  | 0.042 |  |
| CI=Confidence Interval | | All models controlled for age and age^2 | | |  |  |

**(…cont) Table H. Probability for psychiatric distress, for men and women. Models estimated separately by**

**class, LPM.**

|  | **Panel v: Higher non-manual** | | | | | |
| --- | --- | --- | --- | --- | --- | --- |
|  | Unadjusted | | Model 1 | | Model 2 | |
|  | coef. | 95% CI | coef. | 95% CI | coef. | 95% CI |
| Woman | 0.104 | 0.024 - 0.184 | 0.103 | 0.022 - 0.184 | 0.088 | 0.003 - 0.172 |
| Physically strenuous |  |  | 0.058 | -0.012 - 0.128 |  |  |
| Repetitive |  |  | 0.032 | -0.049 - 0.114 |  |  |
| Sitting |  |  | 0.022 | -0.073 - 0.118 |  |  |
| Demand/Control |  |  |  |  | 0.021 | -0.083 - 0.125 |
| Emotional |  |  |  |  | 0.057 | -0.055 - 0.169 |
| Non-flexible |  |  |  |  | -0.121 | -0.248 - 0.005 |
| No errands |  |  |  |  | 0.067 | -0.083 - 0.217 |
| Lacking support |  |  |  |  | 0.091 | -0.086 - 0.269 |
| Constant | -0.081 | -0.784 - 0.622 | -0.152 | -0.857 - 0.552 | -0.12 | -0.813 - 0.573 |
| Observations | 513 |  | 513 |  | 513 |  |
| R-squared | 0.032 |  | 0.039 |  | 0.042 |  |
| CI=Confidence Interval | | All models controlled for age and age^2 | | |  |  |
